# Coordinated Bone–Muscle Axis Association in South China Carp (*Cyprinus carpio rubrofuscus*) with Low Bone Mineral Density: An Integrated Analysis of Muscle Texture, Nutrition, Ultrastructure, and Proteomics

**DOI:** 10.3390/foods15111860

**Published:** 2026-05-24

**Authors:** Kangdi Zhao, Zaixuan Zhong, Jiajia Fan, Yuanyuan Tian, Jun Shi, Dongmei Ma, Huaping Zhu

**Affiliations:** 1 Key Laboratory of Aquatic Animal Immune Technology of Guangdong Province, Pearl River Fisheries Research Institute, Guangzhou 510380, China; zhaokangdi0422@163.com (K.Z.); zhongzaixuan0429@126.com (Z.Z.); fanjiajiaok@163.com (J.F.); tianyuan320@163.com (Y.T.); 2 Department of Aquaculture, College of Marine Sciences, South China Agricultural University, Guangzhou 510642, China; jshi@scau.edu.cn; 3 Key Laboratory of Tropical & Subtropical Fishery Resource Application & Cultivation, Ministry of Agriculture and Rural Affairs, Pearl River Fisheries Research Institute, CAFS, Guangzhou 510380, China

**Keywords:** South China carp, low bone mineral density, bone–muscle axis, proteomics

## Abstract

South China carp (*Cyprinus carpio rubrofuscus*) with low bone mineral density (BMD), characterized by softened bones and wavy ribs, were identified during paddy field aquaculture. This study investigated the association between bone status and muscle traits by comparing muscle texture, nutritional composition, histology, ultrastructure, and proteomic profiles between low-BMD fish (BMD-low, ≤0.02 g/cm^2^) and normal-BMD fish (BMD-normal, ≥0.04 g/cm^2^). Compared with the BMD-normal group, the BMD-low group showed significantly lower muscle resilience and chewiness, significantly higher iron content, and significantly lower methionine and histidine contents (*p* < 0.05). Histological analysis revealed increased muscle fiber density, reduced muscle fiber cross-sectional area, and greater intramuscular lipid accumulation in the BMD-low group (*p* < 0.05). Ultrastructural observations further showed myofibrillar disorganization and elongated sarcomeres and A-bands (*p* < 0.05). Proteomic analysis identified 21 differentially expressed proteins, including up-regulated desmin a and down-regulated nebulin. KEGG enrichment indicated that these proteins were mainly involved in the cytoskeleton in muscle cells, calcium signaling, regulation of actin cytoskeleton, and ubiquitin-mediated proteolysis. These findings provide new insights into the relationship between bone status and muscle quality in South China carp and support future quality evaluation and selective breeding.

## 1. Introduction

Common carp (*Cyprinus carpio*) is one of the most important and oldest cultured freshwater economic fish species with annual output of 2939,315 tons in 2024 [1,2,3]. Now common carps are favored by consumers and cultured in most of China, because of strong environmental adaptability, high survival and growth rates, delicious taste and rich nutrition [4,5]. There are various strains of common carp in different regions of China [6]. South China carp (*Cyprinus carpio rubrofuscus*), one of the four subspecies of the common carp, is native to the Pearl River, Yuanjiang River and the rivers of Hainan Island in South China. It is frequently co-cultured with rice in China, so it is also known as paddy field carp (PF-carp) [7,8].

During the aquaculture of South China carp in paddy fields, we found that some individuals in the population exhibit softened bones and wavy ribs. After being steamed or boiled, the intermuscular bones (IBs) of the carps are too soft to be noticed, so the carps are more favored by the local consumers and farmers. Subsequent X-ray imaging confirmed the low bone mineral density (BMD) characteristic in these carps.

The skeletal system of fish provides structural support, protects internal organs, facilitates movement, and enables adaptation to environmental conditions like temperature, salinity, and pressure [9,10,11]. Bone mineral density (BMD) is a core indicator for assessing bone health in vertebrates. It primarily reflects the mineral content of bone tissue, which is significantly correlated with bone strength and morphology, thereby influencing the realization of its functions [12,13]. Currently, a considerable body of research has been dedicated to fish BMD [14,15]. The skeletal and muscular systems in teleosts remain intrinsically interdependent and tightly coordinated during ontogeny [16,17]. Additionally, the skeletal development of teleosts differs from that of mammals. Most of the human skeleton is endochondral bones, while in Cyprinidae, most parts of the skeleton are intramembranous bones [18,19]. Intermuscular bones (IBs), a structure specific to teleosts, are slender linear structures embedded in muscle. They are formed by direct intramembranous ossification of tendons [20]. In addition, mechanical forces, such as those generated during muscle contractions, play a critical role in shaping bone architecture by stimulating osteoblasts [21,22,23]. However, the relationship between changes in BMD and muscle properties in South China carp remains unclear and warrants further investigation.

In this study, it is speculated that a decrease in skeletal density in South China carp may be accompanied by various changes in muscle properties. A systematic comparison of the muscle characteristics between low bone mineral density (BMD-low) and normal bone mineral density (BMD-nor) groups in the South China carp population was carried out by analyzing muscle texture, histological assessment, nutritional composition, ultrastructure and proteomics. These findings improve our understanding of the bone–muscle relationship in South China carp and provide a basis for the evaluation of muscle quality and future selective breeding.

## 2. Materials and Methods

### 2.1. Fish and Rearing Conditions

A paddy field of about 2000 m^2^ with a rectangular groove (40 cm wide and 50 cm deep) around the field was used to culture South China carps (*Cyprinus carpio rubrofuscus*) in Ruyuan County, Shaoguan City, Guangdong Province. At the end of May 2024, rice seedlings were transplanted in the field with a 10 cm shallow layer of water during and after transplanting. Ten days later, on June 8th, the rice seedlings grew new roots, and about 1500 South China carp fingerlings (average weight 5 g) were transferred to the field. Then the fingerlings were fed on formulated feeds, and the nutritional compositions are listed in detail in Table 1. The formulated feeds were put in the grooves of the field to make up for the shortage of natural diets twice a day in the morning and afternoon. After three months of cultivation, a total of 120 South China carp individuals (120.0 g ± 11.5 g) were collected at random from the paddy fields for the following research.

### 2.2. Bone Mineral Density Analysis and Muscle Sampling

A total of 120 fish were anesthetized in batches with MS222 (Macklin, Shanghai, China), and BMD values of each fish were detected by the X-ray imaging system (Kubtec Xpert 40, Stratford, CT, USA). In order to improve the reliability and accuracy of the results, the most typical individuals were selected in the study. The fish with BMD ≤ 0.02 g/cm^2^ were assigned to the low BMD group (BMD-low group), and the fish with BMD ≥ 0.04 g/cm^2^ were assigned to the normal BMD group (BMD-nor group), while the ones with intermediate BMD values between 0.02 g/cm^2^ and 0.04 g/cm^2^ were not considered in this study.

Then nine fish from each of the BMD-low group and BMD-nor group were randomly selected for further research. Muscle tissues from the corresponding anatomical site of each fish were collected for histology, ultrastructure, muscle texture and proteomic analysis. And the remaining muscle samples were used for the analysis of nutritional composition.

### 2.3. Texture Profile Analysis

Dorsal muscle tissue portions (4 cm × 3 cm × 0.8 cm) were sheared and isolated on ice from the 9 sample fish in each of the two groups for texture profile analysis. Dorsal muscle was collected from the region between the origin of the dorsal fin and the head, above the lateral line. The texture profiles of hardness, springiness, chewiness, cohesiveness, resilience, and shear-force of muscles were determined by a physical property tester (Universal TA, Shanghai, China). The specific parameters were as follows: probe descent rate of 5 mm/s before measurement; probe center descent rate of 2 mm/s; probe return rate of 2 mm/s after measurement; compression degree of 60%; residence interval of 5s; and trigger force of 5 gf [24]. The texture measurement values except shear-force were detected on three different positions of every portion and the average values were calculated, then shear-force was determined only once on one position for each portion.

### 2.4. Analysis of the Nutritional Compositions in Muscle

The dorsal muscle tissues collected randomly from three fish in the same group were pooled as a sample (about 40 g), and there are three mixed samples for each of the two groups. The samples were used to analyze the relationship between muscle nutritional compositions and bone mineral density in South China carps. The nutritional compositions of the dorsal muscle tissues were analyzed following the AOAC standard method (Association of Official Analytical Chemists) [25]. Firstly, muscle proximate composition was determined in terms of moisture, crude protein, crude fat, and ash for each sample. The moisture content of the samples was detected by the freeze-drying method, crude protein content was determined using the Kjeldahl method, crude lipid content was measured by the Soxhlet method, and crude ash was determined by combustion at 550 °C in a muffle furnace [26]. Secondly, the amino acid composition was analyzed. Muscle homogenate was hydrolyzed with 6 M HCl in oxygen-free nitrogen at 110 °C for 22 h.

After the hydrated sample supernatant was filtered with a 0.22 μm membrane filter, amino acids were derivatized by ninhydrin, then compositions were detected using an ultra-high-speed automatic amino acid analyzer (LA8080, Tokyo, Japan).

Thirdly, the fatty acid composition was detected. Muscle samples was mixed with an internal standard solution and hydrolyzed at 80 °C. Subsequently, the hydrolysate was subjected to solvent extraction for lipid removal. After the lipid fraction was saponified to generate fatty acid methyl esters (FAMEs), the samples were extracted and washed with saturated sodium chloride solution. Then the resulting supernatant was filtered and dehydrated. Quantification of fatty acids was performed via gas chromatography with internal standard calibration.

The sample was digested using nitric and perchloric acids following grinding. The contents of three mineral elements (calcium, phosphorus and iron) were then determined using inductively coupled plasma atomic emission spectrometry (ICP-AES). In muscle tissue, calcium and phosphorus are the predominant mineral elements, whereas iron is present as a trace element.

### 2.5. Histological Assessment

Two dorsal muscle samples per fish were collected from nine fish in each of the two groups and fixed in muscle-specific fixative (Servicebio, Wuhan, China) for 24 h at room temperature for further H&E and oil red O staining.

The samples for H&E staining were dehydrated in ethanol, embedded in paraffin, sectioned into 4 µm thick cross sections, stained with hematoxylin–eosin and photographed under a microscope (Nikon, Tokyo, Japan) for general histological observation [27]. Meanwhile, the samples for oil red O staining were dehydrated in 30% sucrose, embedded in O.C.T. compound, and cryosectioned (10 µm). For intermuscular lipid observation, cross sections were stained with oil red O, differentiated in 60% isopropanol, and counterstained with hematoxylin [28]. Five muscle fibers of each slice were selected to measure the long and short diameter. The density of muscle fiber (n/mm^2^) was calculated by counting the fiber number and measuring the Cross-sectional Area (CSA) (mm^2^) of three fields for each slide, and the relative lipid droplet area was quantified using Image-Pro Plus software (version 3.2, Media Cybernetics, Rockville, MD, USA) [27]. Three slides were taken for each sample.

### 2.6. Ultrastructural Observation

Muscle tissue samples (each sample < 1 mm^3^) from three carps of each group were fixed in 2.5% glutaraldehyde and 1% osmium tetroxide. After dehydration in a graded ethanol series (50%, 70%, 80%, and 90%), they were embedded in Epon812 epoxy resin. Ultrathin sections were cut into 70 nm think, stained with 3% uranyl acetate and lead citrate, and examined using a transmission electron microscope (HITACHI7800, Tokyo, Japan). Sarcomere lengths were measured with Image-Pro Plus software (version 3.2).

### 2.7. Proteomic Analysis

#### 2.7.1. Protein Extraction and Digestion of Muscle

The muscle samples (0.5 cm^3^) were separated immediately from the dorsal of fish, and ground into a fine powder in liquid nitrogen. The powdered tissue was then transferred to a 5 mL centrifuge tube for protein extraction. Total proteins were extracted using an 8M urea and the protease inhibitor was added at 10% of the lysate to prevent degradation. The supernatant was collected by centrifugation at 14,100× *g* for 20 min at 4 °C, and then the protein concentration was determined using the Bradford method.

Tryptic digestion was performed as follows. Briefly, a 50 µg aliquot of extracted proteins from each sample was reduced with 200 mM dithiothreitol (DTT) at 37 °C for 1 h. Subsequently, the sample was diluted 8-fold with 50 mM ammonium bicarbonate (ABC) buffer and digested overnight at 37 °C with trypsin, at the enzyme-to-substrate ratio of 1:25 (*w*/*w*).

After digestion, formic acid was added to terminate the reaction. Sequentially, samples were desalted using a C18 desalting column as follows: (1) 100% acetonitrile activated desalting column, 0.1% formic acid balanced column; (2) protein samples were loaded onto the column, and impurities were washed out with 0.1% of formic acid; (3) proteins were eluted by 70% acetonitrile, then collected and freeze-dried.

#### 2.7.2. Liquid Chromatography–Tandem Mass Spectrometry (LC-MS/MS) Analysis

The LC-MS/MS analysis was performed using a nanoflow liquid chromatography system coupled to a mass spectrometer. LC-MS/MS analysis was performed using a Q Exactive HF-X mass spectrometer. Peptide separation was carried out on a 25 cm column (150 μm inner diameter) packed with ReproSil-Pur C18-AQ 1.5-μm silica beads (Beijing Qinglian Biotech Co., Ltd., Beijing, China). A total of 1 μg of peptides was loaded onto the column for each analysis. The mobile phases were composed of (A) 2% methanol and 0.1% formic acid in water, and (B) 0.1% formic acid in 80% acetonitrile. The lyophilized peptide samples were reconstituted in 20 µL of solvent A, clarified by centrifugation (12,000× *g*, 10 min at 4 °C), and the supernatant was subjected to analysis.

For chromatographic separation, peptides were loaded onto a C18 analytical column using the sandwich loading method. A 10 µL aliquot was loaded at a flow rate of 300 nL/min over 15 min. The subsequent analytical separation was conducted at the same flow rate (300 nL/min) for 120 min with the following gradient profile: 4% B was maintained from 0 to 8 min, increased linearly to 10% B by 11 min, further increased to 25% B by 88 min, ramped up to 50% B by 98 min, rapidly increased to 99% B by 102 min, held at 99% B until 108 min, and finally re-equilibrated to the initial conditions.

Label-free quantitative proteomics analysis was performed on a Thermo Orbitrap Fusion mass spectrometer (Thermo Scientific, Waltham, MA, USA). Full MS scans were acquired over a mass range of 250~1450 m/z at a resolution of 120,000 (at 200 m/z). For each cycle, the top 20 most intense precursor ions from the full scan were selected for CID-based MS/MS fragmentation using an isolation window of 1.6 m/z. The normalized collision energy was set to 30% with an activation time of 50 ms. MS/MS spectra were acquired in the linear ion trap using the rapid scan mode, with the following settings: automatic gain control (AGC) target of 7000, maximum injection time of 35 ms, and a dynamic exclusion duration of 18 s.

Subsequently, the acquired raw data files were processed and quantified by Proteome Discoverer software (version 3.2), and searched against the common carp (Cypcar_WageV4.0) database. (1) MS parameters: A Q Exactive HF-X mass spectrometer with a Nanospray Flex™ NSI source was used. Ion spray voltage was 2.2 kV; ion transfer tube temperature was 320 °C. Data-dependent acquisition was performed over m/z 350–1500 (resolution 120,000 at m/z 200; AGC 3 × 10^6^; max injection time 80 ms). The top 40 precursors were fragmented by HCD (resolution 15,000 at m/z 200; AGC 5 × 10^4^; max injection time 45 ms; collision energy 27%). Raw data were saved in .raw format. (2) Proteome Discoverer workflow: The Sequest HT search engine was used with fully tryptic specificity, up to two missed cleavages, and a minimum peptide length of six amino acids. Carbamidomethylation (Cys) was set as a fixed modification and oxidation (Met) as a variable modification. Precursor and fragment mass tolerances were 15 ppm and 0.02 Da, respectively. PSMs and peptides were filtered by Percolator at an FDR < 1%. Protein-level FDR was also controlled at <1%.

#### 2.7.3. Protein Identification and Bioinformatics Analysis

Differential protein abundance between groups was assessed using Student’s *t*-test. Differentially expressed proteins (DEPs) were identified using the following criteria: proteins with a fold change ≥ 2.00 and a *p*-value ≤ 0.05 were considered up-regulated, whereas those with a fold change ≤ 0.50 and a *p*-value ≤ 0.05 were considered down-regulated. Enriched Gene Ontology (GO) terms and Kyoto Encyclopedia of Genes and Genomes (KEGG) pathways were identified from the DEPs using Fisher’s exact test. Functional annotation and enrichment analysis were performed using the R package clusterProfiler (version 3.18.1), with the Gene Ontology (GO) database (https://geneontology.org/) and the Kyoto Encyclopedia of Genes and Genomes (KEGG) database (https://www.kegg.jp/). Data visualization was carried out using ggplot2 (version 3.3.3).

### 2.8. Statistical Analysis

The experimental results are expressed as mean ± standard deviation (mean ± SD). Statistical analysis was performed using an unpaired Student’s *t*-test in R language, with a significance threshold set at *p* < 0.05. Graphs were generated using GraphPad Prism (version 6.02).

## 3. Results

### 3.1. X-Ray Imaging and Bone Density Analysis of South China Carp

**Figure 1:**
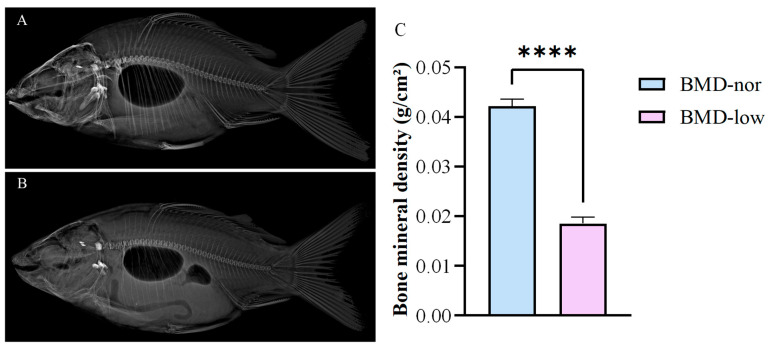
X-ray images and bone mineral density measurements of the South China carp (Cyprinus carpio rubrofuscus) skeletons from normal bone mineral density (BMD-nor) group and low bone mineral density (BMD-low) group. (**A**): X-ray image of South China carp in BMD-nor group; (**B**): X-ray image of South China carp in BMD-low group; (**C**): comparison of average bone mineral densities between the two groups. Significant difference is indicated by “****” (*p* < 0.0001).

Fish individuals (approximately 20%) with the average BMD ≤ 0.02 g/cm^2^ and exhibiting wavy ribs were assigned into the low bone mineral density group (BMD-low), while those (approximately 62%) with BMD ≥ 0.04 g/cm^2^ and smooth ribs were classified into the normal bone mineral density group (BMD-nor) (Figure 1).

### 3.2. Muscle Texture Profile Analysis

The compared results of dorsal muscle texture profiles between the two groups of South China carp indicated that the resilience and chewiness values of the BMD-low group were significantly lower than those of BMD-nor groups (*p* < 0.05) (Table 2). And the values of hardness, cohesiveness and shear-force in the BMD-low group were also lower than those in the BMD-nor group, but the difference was not significant (*p* > 0.05). Only the springiness value of the BMD-low group was slightly higher than that of the BMD-nor group without significant difference (*p* > 0.05).

### 3.3. Comparison of Conventional Nutritional Components and Mineral Content in Muscle

The conventional nutritional component analysis of the South China carps in the two groups is presented in Table 3. It shows that the contents of moisture (74.97 g), crude protein (17.60 g) and ash (1.30 g) in 100 g muscle of BMD-low-group fish were slightly lower than the levels of moisture (75.10 g), crude protein (18.63 g) and ash (1.33 g) in 100 g muscle of BMD-nor-group fish, but the crude fat content of the BMD-low group (6.07 g/100 g) is higher than that of the BMD-nor group (4.80 g/100 g). Meanwhile, all of the differences were not significant (*p* > 0.05).

We compared the levels of three mineral elements in the fish muscle between BMD-nor and BMD-low groups, and the results are summarized in Table 3. Compared with the BMD-nor group, the iron (Fe) content in muscle of BMD-low-group fish was significantly increased (*p* < 0.05), while the phosphorus (P) and calcium (Ca) contents of the muscles from BMD-low group fish were lower than those of the BMD-nor group without significant difference (*p* > 0.05).

### 3.4. Comparison of Amino Acid and Fatty Acid Composition and Content of Dorsal Muscle

The composition and content analysis of amino acids in dorsal muscle is shown in Table 4. The contents of total amino acids (TAA) in South China carp muscles from the BMD-nor group (17.23 g/100 g) and BMD-low group (16.23 g/100 g) had no significant difference. Among essential amino acids (EAA), the methionine and histidine contents of the BMD-low group are significantly lower than that of the BMD-nor group (*p* < 0.05), while no significant differences in contents were detected in the rest of the essential amino acids, including lysine, phenylalanine, isoleucine, leucine, threonine, tryptophan and valine (*p* > 0.05). The comparison of fatty acid contents in the muscle between the BMD-nor and BMD-low group is summarized in Table 5. In the BMD-nor group, the contents of polyunsaturated (PUFAs), saturated (SFAs), and monounsaturated fatty acids (MUFAs) were 0.84, 0.32, and 1.76 g/100g, respectively. The BMD-low group showed higher contents for all three categories—1.21 (PUFAs), 0.49 (SFAs), and 2.96 g/100g (MUFAs)—although the differences were not statistically significant (*p* > 0.05). The BMD-low group exhibited higher contents of oleic acid (C18:1n9), palmitoleic acid (C16:1n7), linoleic acid (C18:2n6), gamma-linolenic acid (C18:3n6), arachidonic acid (C20:4n6), and linolenic acid (C18:3n3) compared to the BMD-normal group (*p* > 0.05).

### 3.5. Muscle Fiber Morphology of Dorsal Muscle

**Figure 2:**
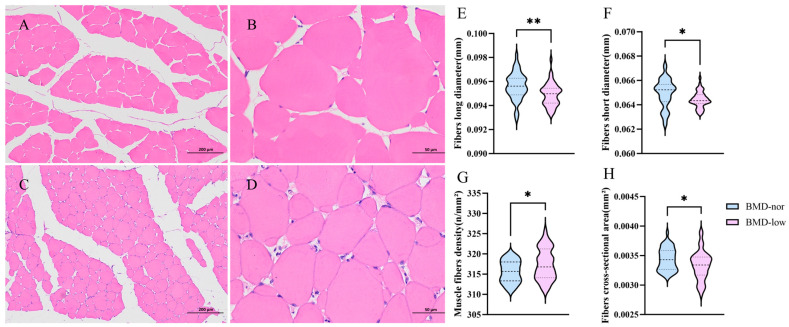
Histological morphology (cross section, hematoxylin and eosin, 40×; 100×) and characteristic of dorsal muscle tissue between BMD-nor and BMD-low. Significant difference between BMD-nor and BMD-low are indicated by “*” (*p* < 0.05), “**” (*p* < 0.01). Scale bars are 200 µm and 50 µm, respectively. (**A**,**B**): The H&E staining of BMD-nor group; (**C**,**D**): the H&E staining of BMD-low group; (**E**): fibers’ long diameter (mm); (**F**): fibers’ short diameter (mm); (**G**): muscle fiber density (n/mm^2^); (**H**): fibers’ cross-sectional area (mm^2^).

To explore whether decreased bone mineral density closely relates to muscle tissue morphology in this study, H&E staining was performed on muscle sections (Figure 2). In both the BMD-nor and BMD-low group, the skeletal muscle fibers were long and columnar, arranged closely and uniformly, with oblong or oval nuclei positioned in the submuscular membrane. Compared with the BMD-nor group, the muscle fibers’ long diameter and short diameter in the BMD-low group decreased remarkably (*p* < 0.05) (Figure 2E,F). Subsequently, in the BMD-low group, the muscle fiber density was higher (*p* < 0.05) and the CSA was significantly lower than the BMD-nor group (*p* < 0.05) (Figure 2G,H).

### 3.6. Lipid Accumulation of Dorsal Muscle

**Figure 3:**
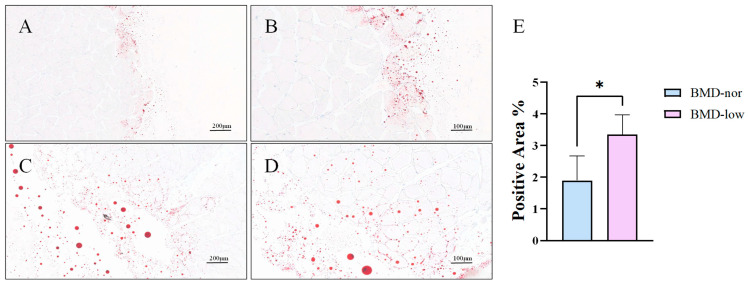
Observation and qualitative analysis of lipid droplet in dorsal muscle tissue between BMD-nor and BMD-low (cross section, oil red O, 40×; 100×). (**A**,**B**): The oil red O staining of muscle fibers in BMD-nor group; (**C**,**D**): the oil red O staining of muscle fibers in BMD-low group; (**E**): Positive Area (%) “*” (*p* < 0.05).

Representative oil red O staining images of the dorsal muscle are shown in Figure 3. Quantitative analysis revealed a significantly higher proportion of stained area in the BMD-low group than in the BMD-nor group (*p* < 0.01) (Figure 3E).

### 3.7. The Ultrastructural Differences in Muscle Fibers Between the Two Groups

**Figure 4:**
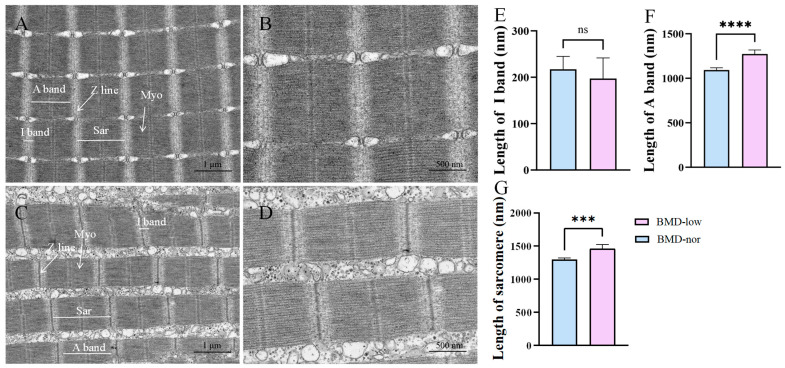
Representative transmission electron micrographs (longitudinal section, 10k×; 30k×) of dorsal muscle sections between BMD-nor and BMD-low. (**A**,**B**): The muscle ultrastructure of BMD-nor-group carp; (**C**,**D**): the muscle ultrastructure of BMD-low-group carp; (**E**): length of A band; (**F**): length of I band; (**G**): length of sarcomere. Note: Sar, sarcomere; Myo, myofilament. *** *p* < 0.001, **** *p* < 0.0001, ns = not significant (*p* ≥ 0.05).

To gain deeper insight differences in muscle fibers between the two groups, transmission electron microscopy (TEM) was applied to examine the ultrastructure of the typical dorsal muscle samples. The longitudinal muscle fiber sections of the dorsal muscles are shown in Figure 4. In the BMD-nor group, the myofibrils are closely organized and the sarcomeres are aligned in a regular pattern. The clear Z-line can be observed in about half of the sarcomeres but disappears in the other half. In the regions of the Z-line, T tubules and terminal cisterns of the sarcoplasmic reticulum forming the intact triads were also observed clearly. In contrast, muscle from the BMD-low group was slightly disorganized in the myofibrils, and misaligned in sarcomeres. But in all of the sarcomeres, the Z-line can be observed clearly. Compared with the BMD-nor group, the BMD-low group displayed wider intermyofibrillar spaces with a swollen sarcoplasmic reticulum. In addition, the irregular T tubules and scattered terminal cisterns made the structures of triads difficult to recognize. Furthermore, we found that the lengths of the sarcomere and A-band in the BMD-low group were significantly longer than those in the BMD-nor group (*p* < 0.05).

### 3.8. Differentially Expressed Protein (DEP) Analysis

In order to uncover the reasons for the differences in muscle properties discovered above, the differentially expressed proteins of dorsal muscle were identified and quantified by a label-free LC-MS/MS proteomics approach. Through label-free analysis, we detected 379,130 MS/MS spectra, identified 20,339 peptides, and discovered 2186 proteins, of which 960 were quantified across the samples.

**Figure 5:**
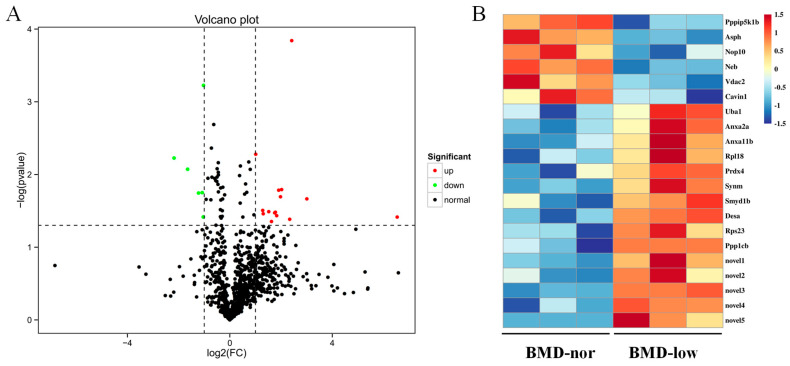
Number and clustering analysis of differentially expressed proteins (DEPs). (**A**): Volcano plot of DEPs between BMD-nor and BMD-low. (**B**): Cluster map showing DEPs between BMD-nor and BMD-low. Note: Red, up-regulated proteins; green, down-regulated proteins; black, non-significantly changed proteins. Columns represent different samples and rows represent different proteins. Clustering was performed using log2-transformed expression values, with red indicating high expression and blue indicating low expression, respectively.

For DEP analysis, the proteins with a fold change ≥ 2.00 and *p*-value ≤ 0.05 were considered a significant up-regulation threshold, while proteins with a fold change ≤ 0.5 and *p*-value ≤ 0.05 were considered a significant down-regulation threshold. And the results showed that, compared with the BMD-nor group, 15 up-regulated and six down-regulated DEPs were identified in the BMD-low group (Figure 5A and Table 6). Among them, compared with the BMD-nor group, in the BMD-low group, diphosphoinositol pentakisphosphate kinase 1b, aspartate beta-hydroxylase, nop10 ribonucleoprotein homolog (yeast), nebulin, voltage-dependent anion channel 2, and caveolae-associated protein 1 were down-regulated, while ubiquitin-like modifier activating enzyme 1, annexin A2a, annexin A11b, ribosomal protein L18, peroxiredoxin 4, synemin (intermediate filament protein), set and mynd domain containing 1b, desmin a, ribosomal protein S23, and protein phosphatase 1 (catalytic subunit, beta isozyme) were up-regulated. Then a hierarchical clustering algorithm was employed to assess the expression patterns within and between groups based on DEPs. The results indicated that within each group, data patterns exhibited high similarity, while between the two groups, distinct differences were observed, which effectively distinguished the groups (Figure 5B).

### 3.9. Functional Annotation of DEPs

Gene Ontology (GO) and Kyoto Encyclopedia of Genes and Genomes (KEGG) pathway were used to functionally annotate the DEPs [29].

**Figure 6:**
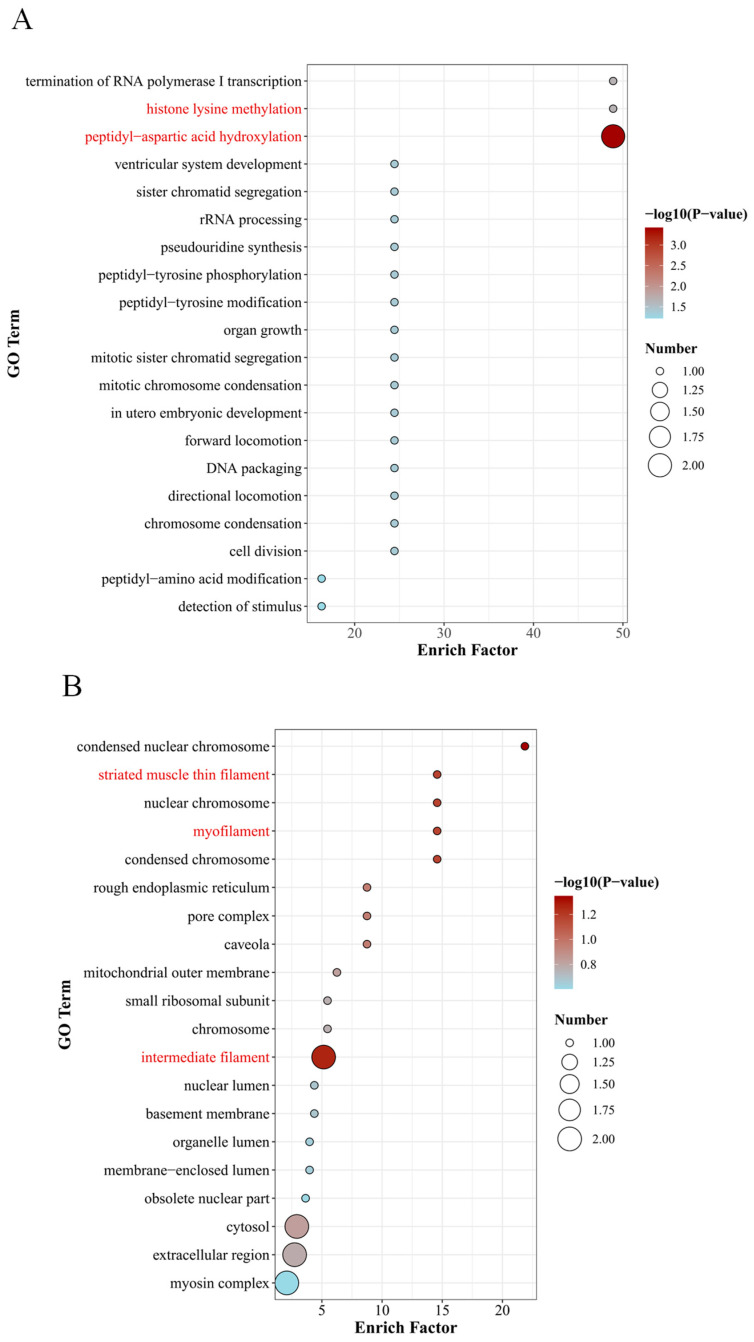

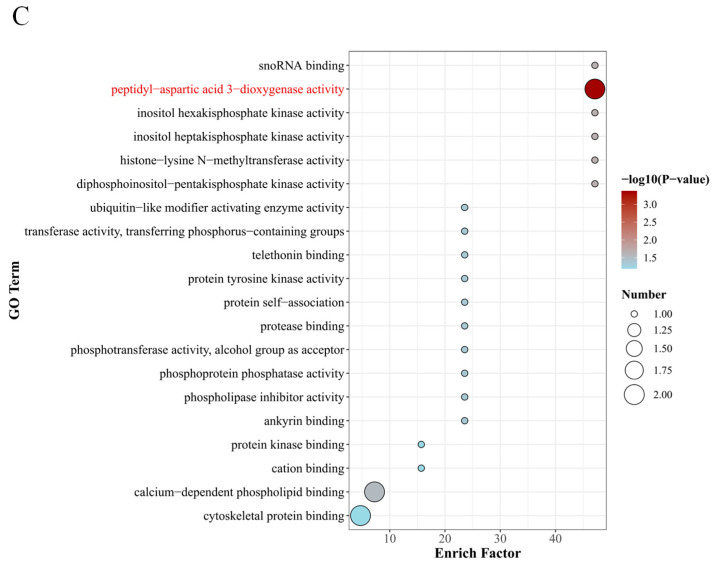
Gene Ontology (GO) annotation statistics of differentially expressed proteins (DEPs) between BMD-nor and BMD-low. (**A**): Biological process (BP) categories of DEPs between BMD-nor and BMD-low. (**B**): Cellular component (CC) categories of DEPs between BMD-nor and BMD-low. (**C**): Molecular function (MF) categories of DEPs between BMD-nor and BMD-low. Note: In this bubble chart, the *x*-axis shows the enrich factor and the *y*-axis lists the GO terms. The size of the dots reflects the number of proteins enriched, and the color intensity represents the significance level.

GO functional annotations are categorized into biological processes, cellular component, and molecular functions (Figure 6). In the comparison between the BMD-nor and BMD-low group, DEPs in the BP are mainly distributed in histone lysine methylation, peptidyl-aspartic acid hydroxylation. In terms of MF, DEPs mainly infect peptidyl-aspartic acid 3-dioxygenase activity. DEPs in CC are significantly enriched in striated muscle thin filament, myofilament, intermediate filament.

**Figure 7:**
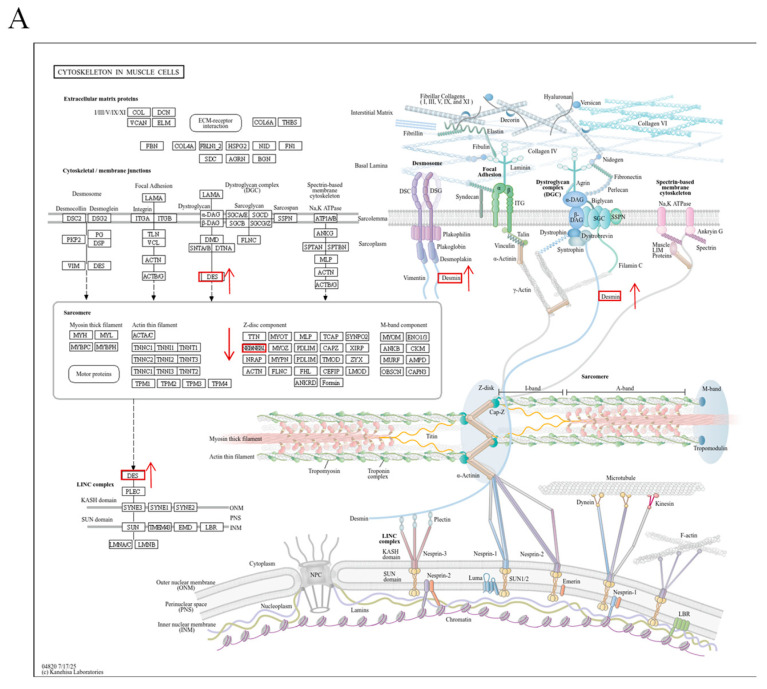

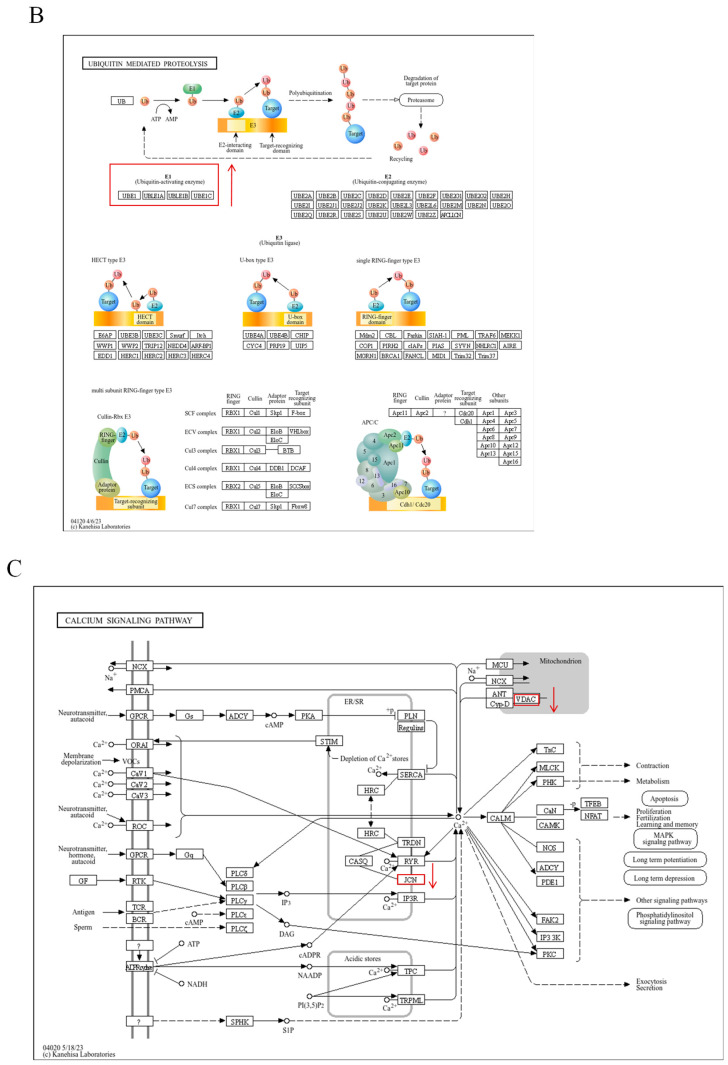

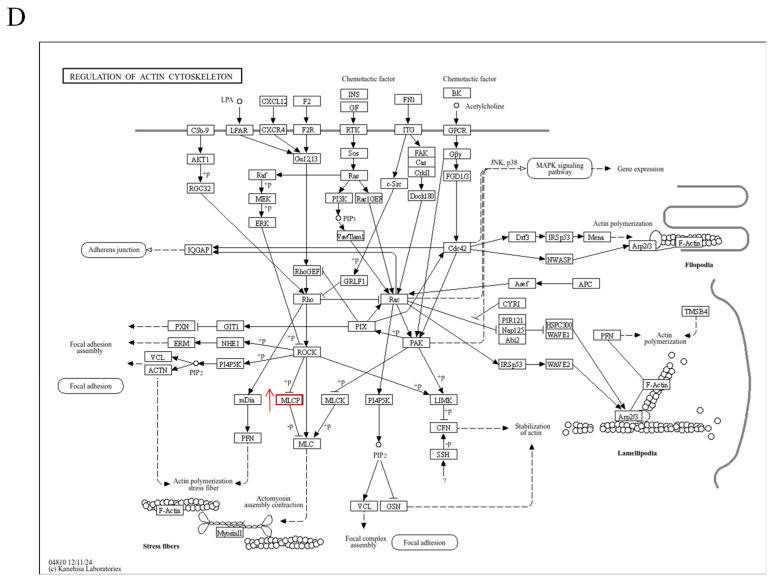
Bubble chart of enrichment of differentially expressed proteins (DEPs) in KEGG pathways between BMD-nor and BMD-low. (**A**): Cytoskeleton in muscle cells; (**B**): ubiquitin-mediated proteolysis; (**C**): calcium signaling pathway; (**D**): regulation of actin cytoskeleton. Red arrow (↑) indicates up-regulated proteins; red arrow (↓) indicates down-regulated proteins.

KEGG pathway analysis showed the DEPs between the two groups were significantly enriched. Protein desmin a and nebulin were enriched in cytoskeleton in muscle cells (ko04820), voltage-dependent anion channel 2 and aspartate beta-hydroxylase were enriched in the calcium signaling pathway (ko04020); protein phosphatase 1, catalytic subunit, beta isozyme was enriched in regulation of actin cytoskeleton (ko04810); ubiquitin-like modifier activating enzyme 1 was enriched in ubiquitin-mediated proteolysis (ko04120) (*p* < 0.05) (Figure 7).

## 4. Discussion

Previous studies have established that bone and muscle are closely linked in structure and function, and prove to be a core unit for movement and metabolism in the organism [30,31]. The present study revealed an association between bone mineral density and alterations in muscle texture properties, myofiber structure, and protein expression in common carp. Proteomic analysis indicates that skeletal muscle participates in this process through some key signaling pathways, for example in the cytoskeleton in muscle cells and regulation of actin cytoskeleton. The findings offer new morphological, mechanical, and molecular evidence for bone–muscle interactions.

In this study, we observed a decrease in muscle fiber cross-sectional area accompanied by an increase in muscle fiber density in the BMD-low group. The results were consistent with the muscle–bone relationship observed in human studies; alterations in muscle fiber size positively correlated with changes in bone mineral density [32,33]. It is known that changes in bone mineral density are linked to the local mechanical environment for muscle attachment and contraction, and this environment is in turn associated with muscle fiber structure [31,34]. This was further confirmed in our study. In this study, ultrastructural observation revealed significant myofilament rearrangement occurred in the BMD-low group, with a longer A-band and sarcomeres. This demonstrates that muscle and bone are inextricably linked in anatomical structure, forming an inseparable functional unit.

In the current study, the differentially expressed protein desmin a was up-regulated in the BMD-low group. Desmin is an intermediate filament protein that plays a pivotal role in maintaining the structural integrity and function of the muscle, ensuring the proper lateral alignment and transmitting mechanical force throughout the cell [35,36,37]. Its higher expression is a well-documented adaptive mechanism in muscle undergoing remodeling or facing mechanical perturbation [38]. Therefore, the up-regulation of desmin a protein observed in the BMD-low group is associated with the structural instability induced by myofilament rearrangement. In addition, the cytoskeleton, with desmin as its core component, directs the assembly and localization of contractile units including myosin filaments [39,40]. First, the reorganized myofibrils and altered cytoskeleton likely impaired contractile force. This would reduce mechanical loading on the bone, reflecting a functional coupling between muscle contractility and bone homeostasis [41,42,43,44]. Second, the altered muscle correlates with a distinct set of secreted factors, and these secreted factors in turn correlate with myokines involved in bone metabolism [45,46]. Collectively, these findings indicated interaction exists between bone and muscle. Nebulin is a skeletal muscle protein that associates with sarcomeric thin filaments and regulates thin filament length and Z-line structure [47]. Compared with the BMD-nor group, nebulin protein was down-regulated in the BMD-low group. This may lead to decreased stability of thin filaments and impaired integrity of the Z-line, thereby contributing to the observed lengthening of the A-band in the BMD-low group. These findings suggest that down-regulation of nebulin is associated with muscle ultrastructural changes associated with reduced bone mineral density, and may represent a potential molecular indicator of this correlation. At the molecular level, these results further support the existence of a close interaction between bone and muscle.

Differentially expressed proteins were significantly enriched in pathways such as the cytoskeleton in muscle cells and regulation of actin cytoskeleton, suggesting that muscle not only serves as an organ of locomotion but also acts as an active endocrine and metabolic regulator [48,49]. This finding is consistent with the classic theory that mechanical forces drive bone adaptation through the cytoskeletal signaling pathway [50]. Among the 21 DEPs identified, several proteins involved in the ubiquitin–proteasome was identified, respectively. The ubiquitin–proteasome system (UPS) is recognized as a major intracellular protein degradation system, and its function is important for muscle homeostasis and health [51]. At the same time, this plays an equally critical role in bone remodeling. Alterations in UPS function have been shown to be linked to both muscle protein degradation pathways and dysregulated bone remodeling pathways, suggesting that UPS may represent a shared correlate between the two tissue systems [52]. This offers a novel perspective for understanding the intrinsic molecular interactions between bone and muscle that extend beyond their traditional mechanical coupling.

In our study, intramuscular fat content was higher in the BMD-low group than in the BMD-nor group. Adipose tissue plays an essential role in skeletal integrity and bone mass. This is because adipocytes, myoblasts, and osteoblasts all share a lineage relationship with mesenchymal stem cells [53]. This interplay is further mediated by endocrine signals from bone itself, such as osteocalcin, which regulates systemic energy metabolism [54]. These studies provide evidence for an intrinsic physiological connection between muscle and bone.

Amino acid analysis revealed that methionine and histidine contents in muscle were significantly decreased in the BMD-low group compared to the BMD-nor group. Methionine is known to play a role in collagen synthesis and bone mineralization [55]. Histidine serves as a precursor for dipeptides such as carnosine and anserine, which are important for antioxidant defense and muscle function [56]. In fish, deficiencies in these amino acids have been associated with reduced muscle quality and skeletal abnormalities [57]. The concurrent reduction in both methionine and histidine observed in the present study may reflect altered protein metabolism in muscle associated with reduced BMD, although the causal direction remains unclear.

Simultaneously, our study found that muscle tissue in the BMD-low group exhibited a significant increase in iron (Fe) content. In fish, nutrient stress is known to trigger a systemic reallocation of resources [58]. Increased iron content is associated with alterations in the endocrine function of muscle [59]. Moreover, iron is an essential trace element for maintaining bone health: it participates in collagen hydroxylation, vitamin D activation, and oxygen transport, with deficiency leading to impaired bone formation [60]. Increased iron content in muscle may disrupt bone metabolic balance via iron-catalyzed generation of reactive oxygen species [61]. This oxidative stress correlates with reduced osteoblastic activity and elevated osteoclastic activity, both of which correlate with decreased bone mineral density [62]. Overall, our data demonstrate that reduced bone mineral density is associated with a compensatory response that involves iron deposition in muscle tissue.

The observed significant reductions in muscle cohesiveness and masticability in the BMD-low group of South China carp have a clear structural basis associated with the microstructural alterations revealed by histological and proteomic analyses. First, histological examination showed that, compared to the BMD-nor group, the BMD-low group exhibited decreased myofiber cross-sectional area and increased myofiber density. This morphological change implies an increased number of intermyofiber contact interfaces per unit area, accompanied by a reduction in individual myofiber diameter. When subjected to masticatory forces, the muscle tissue becomes more prone to separation along the intermyofiber interfaces, thereby manifesting as reduced cohesiveness; the muscle tends to disintegrate easily and lacks elasticity during chewing [63,64]. Second, transmission electron microscopy revealed that, compared to the BMD-normal group, the BMD-low group had significantly increased sarcomere and A-band lengths. The A-band, which corresponds to the myosin filament region, undergoes length changes that reflect reorganization of the contractile unit structure. This structural alteration implies that greater shear deformation is required to disrupt the myofibrils during mastication, thereby increasing the energy required for tissue breakdown. This directly contributes to reduced masticability, requiring more chewing cycles or greater force before swallowing [65]. Third, proteomic analysis provided molecular-level explanations. Down-regulation of nebulin compromises thin filament stability and impairs Z-line integrity, making myofibrils more susceptible to disruption under mechanical stress [66,67]. In contrast, up-regulation of desmin is generally interpreted as a compensatory response to structural instability, indicating impaired lateral alignment of myofibers and compromised mechanical force transmission pathways [68]. The abnormal expression of these two proteins collectively weakens the ability of the muscle cytoskeleton to resist and transmit mechanical forces, ultimately leading to alterations in both muscle cohesiveness and masticability [69,70].

In summary, the multi-level structural alterations—from myofibers to myofibrils to cytoskeletal proteins—collectively constitute the structural basis for the BMD-associated decline in muscle texture properties.

## 5. Conclusions

In this study, the muscle and bone profiles of South China carps (*Cyprinus carpio rubrofuscus*) were compared between the low bone mineral density group (BMD-low group) and the normal bone mineral density group (BMD-nor group) by X-ray imaging, muscle texture detection, histological assessment, nutritional composition determination, TEM observation and proteomic analysis. The results clearly showed significant differences in BMD, resilience and chewiness of muscle texture, iron (Fe) content, methionine and histidine contents, muscle fiber density, cross-sectional area, oil red O staining level, organizational and arrangement patterns of muscle fiber, and the proteome. In conclusion, our findings reveal coordinated bone–muscle crosstalk in South China carp, linking bone mineral density to multiple muscle profiles and providing evidence for the intrinsic interplay between skeletal muscle and bone during ontogeny.

## Figures and Tables

**Table 1:** Ingredients and proximate compositions of the diets for South China carp cultured in paddy fields.

Ingredient Composition	Content (%)
fish meal	10.00
soybean meal	10.00
peanut meal	25.00
cottonseed meal	12.00
rapeseed meal	10.00
wheat flour	15.00
rice bran	12.00
fish oil	3.00
vitamin complex	1.00
composite mineral	2.00
total	100.00
proximate composition	
crude protein	33.64
crude lipid	5.80
crude ash	4.30

**Table 2:** Comparison of the texture profiles of South China carp muscle between BMD-low and BMD-nor.

Items	BMD-Nor	BMD-Low
Hardness (N)	195.34 ± 17.98	161.12 ± 12.86
Springiness (mm)	0.57 ± 0.01	0.59 ± 0.03
Resilience	0.91 ± 0.03 ^a^	0.77 ± 0.02 ^b^
Chewiness (mJ)	76.94 ± 6.06 ^a^	61.20 ± 2.26 ^b^
Cohesiveness	0.65 ± 0.00	0.64 ± 0.02
Shear-force (N)	2450.79 ± 435.80	2386.12 ± 297.63

Note: Data are presented as mean ± SD (n = 9). Different letters in the same row indicate significant differences (*p* < 0.05).

**Table 3:** Comparison of proximate composition and mineral content between BMD-low and BMD-nor of South China carp muscle (g/100g).

Items	BMD-Nor	BMD-Low
moisture	75.1 ± 0.85	74.97 ± 0.59
ash	1.33 ± 0.06	1.30 ± 0.10
crude protein	18.63 ± 0.61	17.6 ± 1.13
crude fat	4.80 ± 1.31	6.07 ± 0.95
phosphorus	0.21 ± 0.02	0.21 ± 0.00
calcium	0.04 ± 0.08	0.03 ± 0.02
iron	0.005 ± 0.00 ^a^	0.006 ± 0.001 ^b^

Note: Data are presented as mean ± SD (n = 3). Different letters in the same row indicate significant differences (*p* < 0.05).

**Table 4:** Comparison of amino acid content in dorsal muscle of South China carp between BMD-nor and BMD-low (g/100 g).

Items	BMD-Nor	BMD-Low
aspartic acid (asp) ^§^	1.89 ± 0.05	1.79 ± 0.09
threonine (thr) *	0.85 ± 0.02	0.80 ± 0.04
serine (ser) ^§^	0.75 ± 0.02	0.72 ± 0.04
glutamic acid (glu) ^#§^	2.87 ± 0.09	2.71 ± 0.14
glycine (gly) ^§^	0.86 ± 0.04	0.87 ± 0.04
alanine (ala) *^§^	1.13 ± 0.03	1.07 ± 0.05
valine (val)	0.90 ± 0.02	0.85 ± 0.03
methionine (met) *	0.31 ± 0.03 ^a^	0.19 ± 0.05 ^b^
isoleucine (ile) *	0.83 ± 0.02	0.78 ± 0.04
leucine (leu )*	1.45 ± 0.04	1.38 ± 0.08
tyrosine (tyr) ^#§^	0.56 ± 0.03	0.53 ± 0.04
phenylalanine (phe) *	0.79 ± 0.02	0.76 ± 0.03
lysine (lys) *	1.74 ± 0.05	1.65 ± 0.09
histidine (his) *^#^	0.63 ± 0.01 ^a^	0.50 ± 0.08 ^b^
arginine (arg) ^#^	1.05 ± 0.02	1.01 ± 0.08
proline (pro) ^§^	0.61 ± 0.02	0.60 ± 0.04
TAA	17.23 ± 0.38	16.23 ± 0.86
EAA	7.50 ± 0.21	6.91 ± 0.44
SEAA	5.11 ± 0.15	4.75 ± 0.34
DAA	8.11 ± 0.25	8.17 ± 0.40

Note: * indicates essential amino acids (EAAs); # indicates semi-essential amino acids (SEAA); § indicates delicious. Different letters in the same row indicate significant differences (*p* < 0.05).

**Table 5:** Comparison of fatty acid content in dorsal muscle of South China carp between BMD-nor and BMD-low (g/100 g).

Items	BMD-Nor	BMD-Low
myristic acid (C14:0)	0.03 ± 0.01	0.04 ± 0.01
pentadecanoic acid (C15:0)	0.01 ± 0.01	0.01 ± 0.01
palmitic acid (C16:0)	0.00 ± 0.01	0.00 ± 0.01
palmitoleic acid (C16:1)	0.48 ± 0.20	0.77 ± 0.15
stearic acid (C18:0)	0.13 ± 0.07	0.21 ± 0.03
oleic acid (C18:1)	1.28 ± 0.54	2.19 ± 0.49
arachidic acid (C20:0)	0.15 ± 0.05	0.22 ± 0.05
gondoic acid (C20:1)	0.06 ± 0.02	0.09 ± 0.01
eicosadienoic acid (C20:2n-6)	0.02 ± 0.01	0.02 ± 0.01
behenic acid (C22:0)	ND	0.01 ± 0.01
linoleic acid (C18:2n-6)	0.66 ± 0.28	1.01 ± 0.16
gamma-linolenic acid (C18:3n-6)	0.02 ± 0.01	0.02 ± 0.01
alpha-linolenic acid (C18:3n-3)	0.03 ± 0.01	0.04 ± 0.01
di-γ-linolenic acid (C20:3n-6)	0.03 ± 0.01	0.04 ± 0.01
arachidonic acid (C20:4n-6)	0.08 ± 0.02	0.10 ± 0.01
eicosapentaenoic acid (C20:5n-3)	0.01 ± 0.01	0.01 ± 0.01
docosahexaenoic acid (C22:6n-3)	0.04 ± 0.01	0.03 ± 0.01
∑SFA	0.32 ± 0.15	0.49 ± 0.12
∑MUFA	1.82 ± 0.76	3.05 ± 0.65
∑PUFA	0.13 ± 0.05	0.14 ± 0.05
∑(n-3) PUFA	0.08 ± 0.03	0.08 ± 0.03
∑(n-6) PUFA	0.05 ± 0.02	0.06 ± 0.02

Note: SFA, saturated fatty acids; MUFA, monounsaturated fatty acids; PUFA, polyunsaturated fatty acids.

**Table 6:** Differentially expressed proteins in dorsal muscle of South China carp between BMD-nor and BMD-low.

ID	Name	log2FC	*p*-Value
Down-regulated			
ENSCCRP00000035177.2	Diphosphoinositol pentakisphosphate kinase 1b	−2.17	0.01
ENSCCRP00000107090.1	Aspartate beta-hydroxylase	−1.65	0.01
ENSCCRP00000146052.1	NOP10 ribonucleoprotein homolog (yeast)	−1.22	0.02
ENSCCRP00000162534.1	Nebulin	−1.03	0.00
ENSCCRP00000116463.1	Voltage-dependent anion channel 2	−1.08	0.02
ENSCCRP00000116664.1	Caveolae associated protein 1	−1.04	0.04
Up-regulated			
ENSCCRP00000090147.2	Ubiquitin-like modifier activating enzyme 1	1.63	0.04
ENSCCRP00000160900.1	Annexin A2a	2.34	0.04
ENSCCRP00000113720.1	Annexin A11b	1.75	0.03
ENSCCRP00000010581.2	Ribosomal protein L18	1.75	0.03
ENSCCRP00000162781.1	Peroxiredoxin 4	1.31	0.03
ENSCCRP00000021960.2	Synemin, intermediate filament protein	1.98	0.02
ENSCCRP00000066707.1	SET and MYND domain containing 1b	1.28	0.03
ENSCCRP00000064266.1	Desmin a	1.01	0.01
ENSCCRP00000179338.1	Ribosomal protein S23	1.91	0.02
ENSCCRP00000181581.1	Protein phosphatase 1, catalytic subunit, beta isozyme	1.83	0.04
ENSCCRP00000171729.1	Novel	1.78	0.03
ENSCCRP00000106251.1	Novel	2.42	0.00
ENSCCRP00000025911.2	Novel	2.02	0.02
ENSCCRP00000174858.1	Novel	6.54	0.04
ENSCCRP00000123044.1	Novel	3.01	0.02

## Data Availability

The original contributions presented in this study are included in the article. Further inquiries can be directed to the corresponding authors.
